# A rare clinical image of strawberry nevi of infancy

**DOI:** 10.11604/pamj.2024.49.22.42821

**Published:** 2024-09-27

**Authors:** Minal Dambhare, Archana Mourya

**Affiliations:** 1 Department of Paediatric Nursing Smt, Radhikabai Meghe Memorial College of Nursing, Datta Meghe Institute of Higher Education and Research (Deemed to be University), Sawangi Wardha, Maharashtra, India

**Keywords:** Strawberry nevi, infancy, capillary hemangioma, benign orbital neoplasms, angioembolization

## Image in medicine

Strawberry nevi of infancy are clusters of extra blood vessels on a baby's skin. It may be present on any site of the body. It was observed at one month of age. Strawberry nevi of infancy constitute 8-10% of benign tumors in the pediatric age group, 80% of which occur in the head and neck region. They are more common in females with a female-to-male ratio of 3:2 to 5:1. A 7-month-old female baby presented to our clinic with a noticeable mass over the right eye since birth, swelling on the right eye, and red mass over the right eye, also called strawberry birthmark. The cause is unknown. A physical examination is usually sufficient to diagnose strawberry nevi of infancy. The physical examination results showed a strawberry mass present over the right eyes and watery eyes, and mucopurulent discharge present. Mass initially bigger in size and not able to open right eyes properly. An investigation of CEMRI BRAIN and ORBIT findings shows intensely enhancing altered signal intensity lesion in the right periorbital region with extension in the extra-conal, conal compartment with no involvement of intraconal compartment and brain parenchyma. The lesion involves the superior rectus, superior oblique, levator palpebrae superioris, and subcutaneous plane of preseptal region, medial and lateral canthus region, right lacrimal gland, and measuring 4.2 x 3.7 x 3.6 cm. The lesion appears isointense on T1, iso to hyperintense on T2/FLAIR with no restriction on diffusion weighted imaging (DWI) with multiple serpiginous flow voids. Mucosal thickening was noted in the left mastoid. Treatment has been given corticosteroids and beta-blockers. Treatment of corticosteroids and beta-blockers was given and the patient was advised to plan for angioembolization.

**Figure 1:**
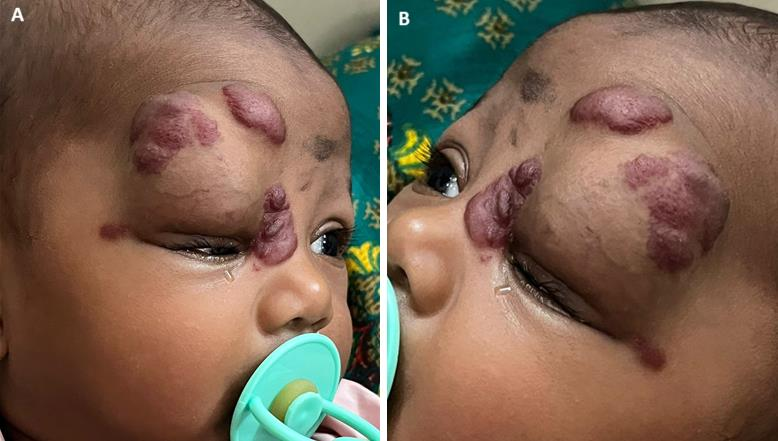
A) strawberry mass over the right eyebrows and right periorbital region; B) strawberry mass over the right eye, swelling over the eyelids and watery eyes

